# Photoconductivities in monocrystalline layered V_2_O_5_ nanowires grown by physical vapor deposition

**DOI:** 10.1186/1556-276X-8-443

**Published:** 2013-10-25

**Authors:** Ruei-San Chen, Wen-Chun Wang, Ching-Hsiang Chan, Hung-Pin Hsu, Li-Chia Tien, Yu-Jyun Chen

**Affiliations:** 1 Graduate Institute of Applied Science and Technology, National Taiwan University of Science and Technology, 43, Sec.4, Keelung Rd., Taipei 10607, Taiwan; 2 Department of Electronic Engineering, National Taiwan University of Science and Technology, Taipei 10607, Taiwan; 3 Department of Electronic Engineering, Ming Chi University of Technology, Taishan, Taipei 243, Taiwan; 4 Department of Materials Science and Engineering, National Dong Hwa University, Shoufeng, Hualien 974, Taiwan

**Keywords:** Vanadium pentoxide, Nanowire, Photoconductivity, Physical vapor deposition, Normalized gain

## Abstract

Photoconductivities of monocrystalline vanadium pentoxide (V_2_O_5_) nanowires (NWs) with layered orthorhombic structure grown by physical vapor deposition (PVD) have been investigated from the points of view of device and material. Optimal responsivity and gain for single-NW photodetector are at 7,900 A W^-1^ and 30,000, respectively. Intrinsic photoconduction (PC) efficiency (i.e., normalized gain) of the PVD-grown V_2_O_5_ NWs is two orders of magnitude higher than that of the V_2_O_5_ counterpart prepared by hydrothermal approach. In addition, bulk and surface-controlled PC mechanisms have been observed respectively by above- and below-bandgap excitations. The coexistence of hole trapping and oxygen sensitization effects in this layered V_2_O_5_ nanostructure is proposed, which is different from conventional metal oxide systems, such as ZnO, SnO_2_, TiO_2_, and WO_3_.

## Background

Vanadium pentoxide (V_2_O_5_) is the most stable crystallization form and is also the most applicable in the industry among vanadium oxide systems such as VO, VO_2_, and V_2_O_3_. The orthorhombic layered structure of V_2_O_5_ promises a high ionic storage capacity for energy storage applications [1]. Recently, its quasi-one-dimensional nanostructures such as nanowires (NWs), nanobelts (NBs), and nanotubes have gained substantial attention. Due to high surface-to-volume ratio and high surface activity, V_2_O_5_ 1D structures for various applications, such as field emitters [2-5], transistors [6,7], chemical sensors [8-10], and lithium batteries [11-14], have been developed.

In addition, V_2_O_5_ with a direct optical bandgap at visible-light region (*E*_g_ = 2.2 to 2.7 eV) [2,15-18] also inspires the studies of optoelectronic applications such as photodetection [2,19], optical waveguide [20], and high-speed photoelectric switch [21]. Although device performance of the individual NW has been demonstrated in several studies, fundamental photoconduction (PC) properties and their corresponding surface effects were less studied than the known hopping transport [6,21-24]. The potential difference of the transport properties of nanomaterials grown by different approaches was also less known. In this paper, we report the study of photoconductivities of V_2_O_5_ NWs grown by physical vapor deposition (PVD). The performance of the single-NW device and intrinsic PC efficiency of the material have been defined and discussed. The results are also compared with the reported data of the V_2_O_5_ counterpart synthesized by hydrothermal approach. The probable PC mechanisms that originated from the bulk and surface under above- and below-bandgap excitations are also proposed.

## Methods

V_2_O_5_ NWs were grown by PVD using high-purity V_2_O_5_ powder as the source material and mixed O_2_/Ar as the carrier gas. The growth temperature was 550°C, and the pressure was 0.3 Torr. The details of material growth can be found in our earlier publications [25,26]. The morphology, structure, and crystalline quality of the as-grown V_2_O_5_ NWs were characterized by field-emission scanning electron microscopy (FESEM), X-ray diffraction (XRD), Raman spectroscopy, high-resolution transmission electron microscopy (HRTEM), and selected-area electron diffraction (SAD). Electrical contacts of the two-terminal single-NW devices were fabricated by focused ion beam (FIB; FEI Quanta 3D FEG, FEI Company, Hillsboro, OR, USA) deposition using platinum (Pt) as the metal electrode. Individual NWs were dispersed on the insulating Si_3_N_4_/*n*-Si or SiO_2_/*n*-Si template with pre-patterned Ti/Au microelectrodes prior to FIB deposition. Electrical measurements were carried out on an ultralow-current leakage cryogenic probe station (TTP4, LakeShore Cryotronics, Inc., Westerville, OH, USA). A semiconductor characterization system (4200-SCS, Keithley Instruments Inc., Cleveland, OH, USA) was utilized to source dc bias and measure current. He-Cd gas laser and diode laser were used to source excitation lights with wavelengths (*λ*) at 325 and 808 nm for the PC measurements, respectively. The incident power of laser was measured by a calibrated power meter (Ophir Nova II, Ophir Optronics, Jerusalem, Israel) with a silicon photodiode head (Ophir PD300-UV). A UV holographic diffuser was used to broaden laser beam size (approximately 20 mm^2^) to minimize error in power density calculation.

## Results and discussion

**Figure 1:**
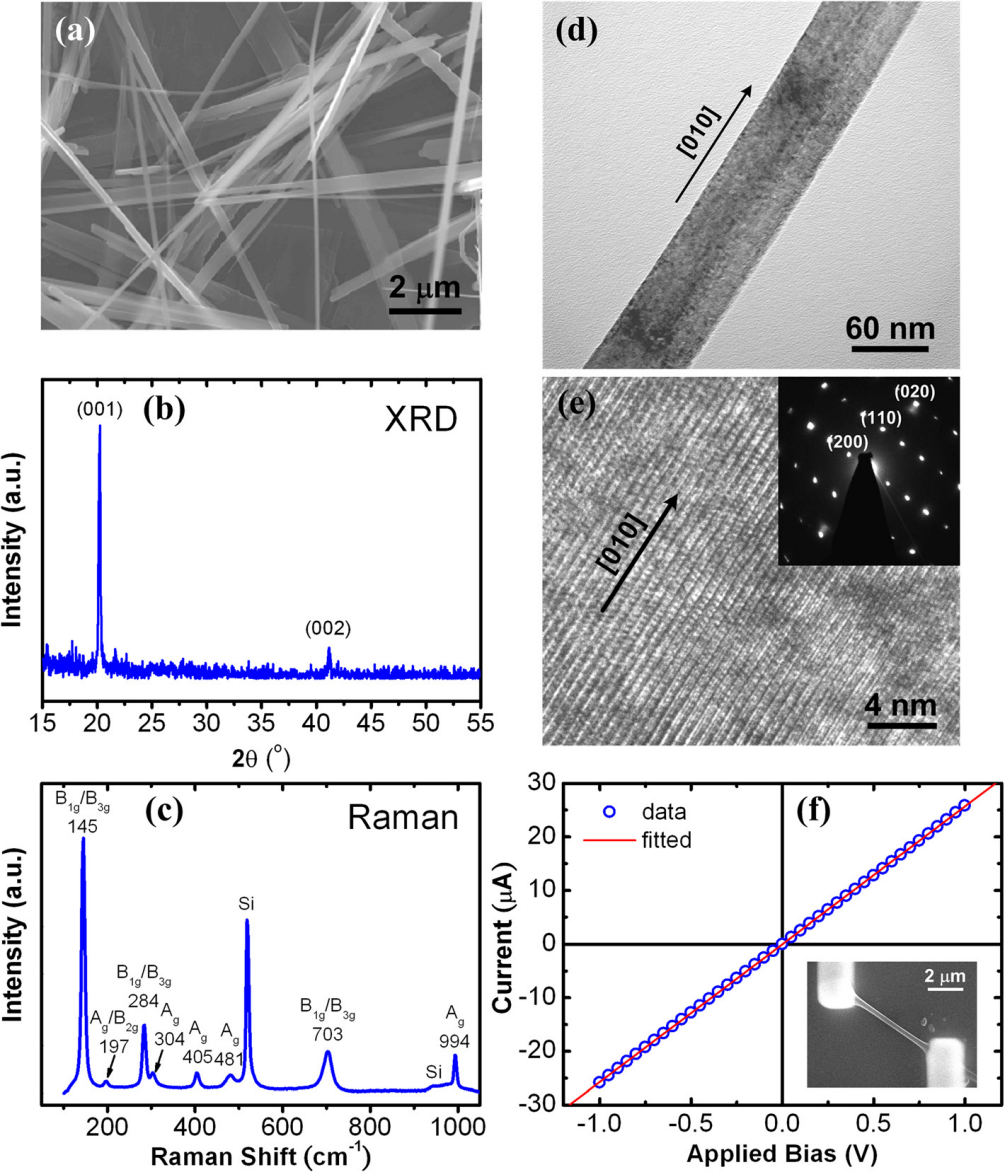
**FESEM, TEM, and HRTEM images, XRD and SAD patterns, Raman spectrum, and *i***_**d**_**-*V *measurement of V**_**2**_**O**_**5 **_**NW. (a)** FESEM image, **(b)** XRD pattern, **(c)** Raman spectrum of the ensembles of V_2_O_5_ NWs grown by PVD. **(d)** TEM image and corresponding **(e)** HRTEM image and SAD pattern focused on an individual V_2_O_5_ NW. **(f)** Dark current versus applied bias measurement in air ambience for single V_2_O_5_ NW with *d* = 400 ± 50 nm and *l* = 7.3 μm. A typical FESEM image of the single V_2_O_5_ NW device fabricated by FIB approach is also shown in the inset of **(f)**.

A typical FESEM image of V_2_O_5_ NW ensembles grown as described above on silicon substrate prepared by PVD is shown in Figure 1a. The micrograph reveals partial V_2_O_5_ 1D nanostructures with slab-like morphology. The diameter (*d*), which is defined as the width of the NWs with relatively symmetric cross section, is in the range of 100 to 800 nm. The length usually is longer than 10 μm. The XRD pattern shows the predominant diffraction peaks at 20.3° and 41.2° (Figure 1b), which is consistent with the (001) and (002) orientations of the orthorhombic structure (JCPDS no. 41–1426). The Raman spectrum shows the eight signals at positions of 145 cm^-1^ (B_1g_/B_3g_), 197 cm^-1^ (A_g_/B_2g_), 284 cm^-1^ (B_1g_/B_3g_), 304 cm^-1^ (A_g_), 405 cm^-1^ (A_g_), 481 cm^-1^ (A_g_), 703 cm^-1^ (B_1g_/B_3g_), and 994 cm^-1^ (A_g_), which correspond to the phonon modes in previous reports [17,27,28], further confirming the orthorhombic crystalline structure of the V_2_O_5_ NWs (Figure 1c). Two major Raman peaks at low-frequency positions of 145 and 197 cm^-1^ that originated from the banding mode of (V_2_O_2_)_*n*_ also indicate the long-range order layered structure of V_2_O_5_ NWs. In addition, the single-crystalline quality of the V_2_O_5_ NWs is further confirmed by the TEM and SAD measurements. Figure 1d shows the TEM image focused on an individual V_2_O_5_ NW. The clear lattice image can be observed by HRTEM as depicted in Figure 1e. The preferential growth orientation of long axis along 〈010〉 is also confirmed by the corresponding SAD pattern with zone axis along 〈001〉 as shown in the inset of Figure 1e [12].

Electrical contacts of single V_2_O_5_ NW devices were examined by dark current versus applied bias (*i*_d_-*V*) measurements. Figure 1f depicts typical *i*_d_-*V* curves measured at room temperature of 300 K for the V_2_O_5_ NW with *d* at 400 ± 50 nm and the inter-distance between two contact electrodes (*l*) at 7.3 μm. A representative FESEM image of the individual V_2_O_5_ NW device is also shown in the inset of Figure 1f. The *i*_d_-*V* curve reveals a linear relationship, indicating the ohmic contact condition of the NW device. Room temperature conductivity (*σ*) was estimated at 13 ± 3 Ω^-1^ cm^-1^. A similar *σ* can be reproduced from the other samples with a *d* range of 200 to 800 nm. The *σ* level is more than one order of magnitude higher than that (*σ* = 0.15 to 0.5 Ω^-1^ cm^-1^) of individual V_2_O_5_ NWs in previous reports in which small polaron hopping is attributed to the transport mechanism [23,24].

**Figure 2:**
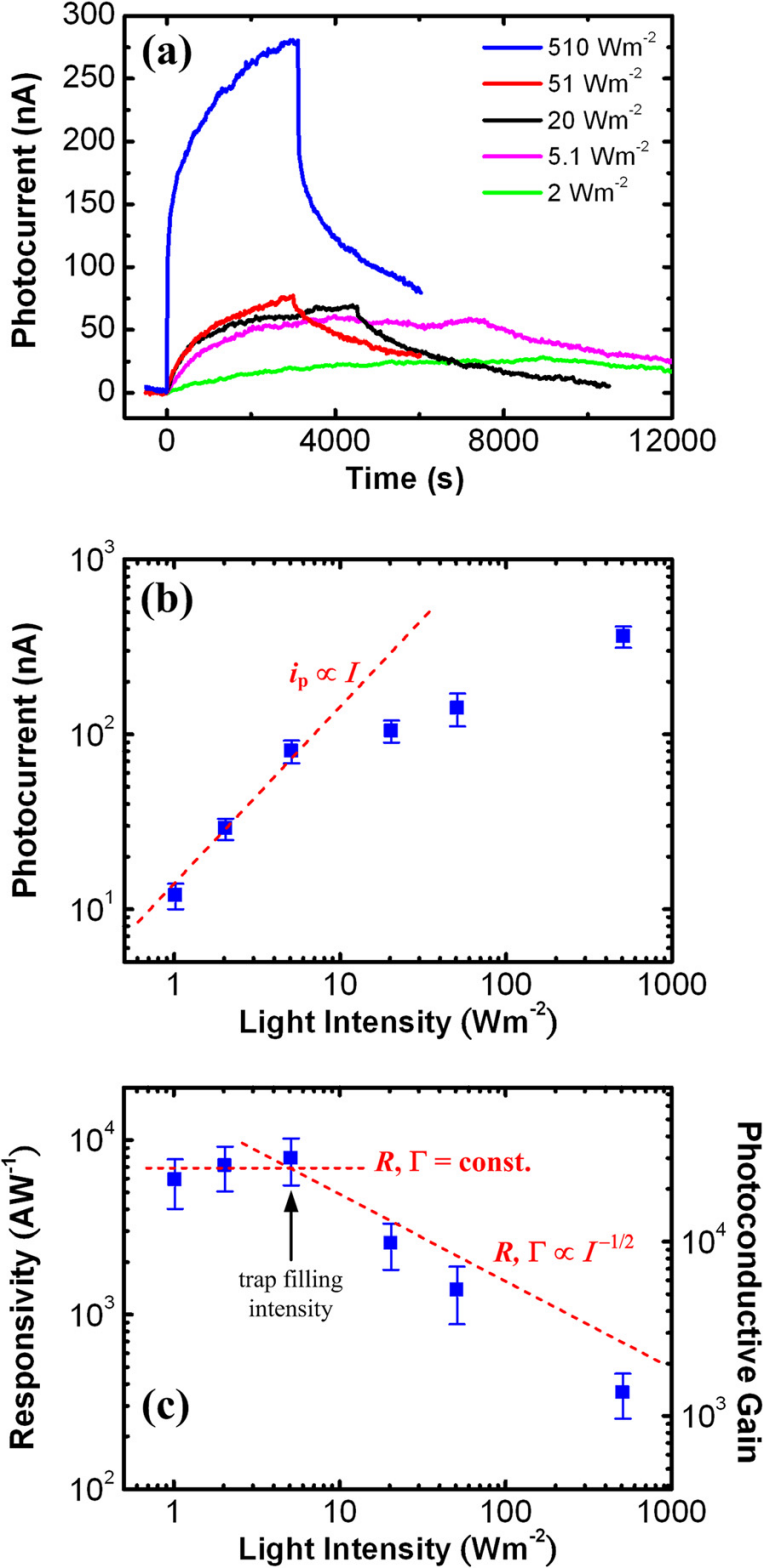
**Photocurrent response curves, estimated photocurrent versus intensity, and calculated responsivity and gain versus intensity. (a)** Photocurrent response curves under inter-band excitation (*λ* = 325 nm) with different intensities, **(b)** estimated steady-state photocurrent versus intensity, and **(c)** calculated responsivity and gain versus intensity measured at a bias of 0.1 V for the V_2_O_5_ NW with *d* = 800 nm and *l* = 2.5 μm.

The photocurrent response curves for the 325-nm band-to-band excitation under different light intensity (*I*) at a bias of 0.1 V for the V_2_O_5_ NW with *d* = 800 nm and *l* = 2.5 μm are illustrated in Figure 2a. A constant background current has been subtracted to reveal the photocurrent values. The result shows that the photoresponse takes a rather long time to reach a steady state. The estimated steady-state photocurrent (*i*_p_) versus *I* is plotted in Figure 2b. The *i*_p_ shows a linear increase with the increase of *I* below a critical power density at approximately 5 W m^-2^. Once *I* exceeds the critical value, the *i*_p_ deviates from the linear behavior and appears to saturate gradually. To investigate the device performance and PC mechanism underneath the power-dependent *i*_p_, two quantities, namely responsivity (*R*) and photoconductive gain (Γ) which determine the photodetector performance, will be defined and discussed.

The responsivity *R* is defined as the photocurrent generated by the power of light incident on an effective area of photoconductor, i.e.,

$$R=\frac{i_{p}}{P_{\mathrm{NW}}},$$

where *P*_NW_ is the incident optical power on the projected area (*A*) of the measured NW and can be calculated as *P*_NW_*= IA = Idl*[29]*]*. The calculated *R* versus *I* result according to the measured *i*_p_ values in Figure 2b is depicted in Figure 2c. The result shows that *R* increases from 360 to 7,900 A W^-1^ gradually and saturates at a near-constant level while intensity decreases from 510 to 1 W m^-2^. While comparing the optimal *R* with that of earlier reports, the value at 7,900 A W^-1^ is over one order of magnitude higher than that (*R* ~ 482 A W^-1^) of V_2_O_5_ NWs synthesized by hydrothermal approach [2]. Even if the comparison is made at similar power densities in the range 20 to 30 W m^-2^, the PVD-grown V_2_O_5_ NW still exhibits higher *R* at approximately 2,600 than the reference data by a factor of 5. In addition, compared to other nanostructured semiconductor photodetectors, the *R* of the V_2_O_5_ NW device is higher than those of ZnS NBs (*R* ~ 0.12 A W^-1^) [30], ZnSe NBs (*R* ~ 0.12 A W^-1^) [31], ZnO nanospheres (*R* ~ 14 A W^-1^) [32], and Nb_2_O_5_ NBs (*R* ~ 15 AW^-1^) [33] and is lower than those of GaN NWs (*R* ~ 10^6^ A W^-1^) [34] and ZnS/ZnO biaxial NBs (*R* = 5 × 10^5^ A W^-1^) [35].

To investigate the Γ which is a physical quantity determining the photocarrier collection efficiency of a photodetector, Γ is estimated according to its linear relationship with *R* and *i*_p_, i.e.,

$$\Gamma =\frac{E}{e}\;\frac{R}{\eta }=\frac{E}{e}\frac{i_{p}}{\mathrm{\eta P}},$$

where *E* is the photon energy, *e* is the elementary electron charge, and *η* is the quantum efficiency [29]. To simplify the calculation, the *η* is assumed to be unity. The calculated Γ versus *I* result is also plotted in Figure 2c. The maximal Γ of this work at approximately 3 × 10^4^ is also over one order of magnitude higher than that (Γ = 1328) of the hydrothermal-synthesized V_2_O_5_ NWs [2]. Compared with other nanostructured semiconductor devices, the Γ of the V_2_O_5_ NW is higher than those of ZnS NBs (Γ ~ 0.5 A W^-1^) [30], ZnSe NBs (Γ ~ 0.4 A W^-1^) [31], ZnO nanospheres (Γ ~ 5 A W^-1^) [32], Nb_2_O_5_ NBs (Γ ~ 6 A W^-1^) [33], and WO_3_ NWs (Γ ~ 5×10^3^ A W^-1^) [36] and is lower than those of ZnO NWs (Γ ~ 2 × 10^8^ A W^-1^) [37], SnO_2_ NWs (Γ ~ 9 × 10^7^ A W^-1^) [38], GaN NWs (Γ ~ 10^6^ A W^-1^) [34], and ZnS/ZnO biaxial NBs (Γ = 2 × 10^6^ A W^-1^) [35].

In addition, the power-dependent behavior of *R* (or Γ) could imply the potential hole trapping PC mechanism. The unintentionally doped V_2_O_5_ semiconductor has been confirmed to exhibit n-type conducting [6,22,39]. Under low power density, the photoexcited holes are totally captured by certain defects which function as a hole trap. The hole trapping effect leaves unpaired electrons which exhibit a long lifetime (*τ*). As photocurrent is linearly dependent on carrier lifetime, i.e., *i*_p_ ∝ *τ*, the long-lifetime electron will substantially enhance and dominate the photocurrent generation. As the *τ* of electron which is decided by the hole trapping time is now a constant, *R* (or Γ) will be independent of the excitation power, i.e., *R* (or Γ) = const. Once the power exceeds a critical value (trap filling intensity), the photogenerated hole density is much higher than the trap density and the traps will be fully occupied. Under this condition, the trapping effect can be ignored and photocarriers will follow the bimolecular recombination mechanism [40-42]. The recombination after trap filling results in the decrease of *τ* with the increase of *I*, making an intensity-dependent *R* (or Γ) following an inverse power law, i.e*.*, *R* (or Γ) ∝ *I*^-*k*^, where the theoretical *k* = 1/2 [42]. The aforementioned model agrees with the two-stage power-dependent *R* (or Γ) result in Figure 2c and *i*_p_ in Figure 2b. The trap filling intensity is roughly at 5 W m^-2^, and the fitted *k* value is 0.62 ± 0.04 for the V_2_O_5_ NWs.

**Figure 3:**
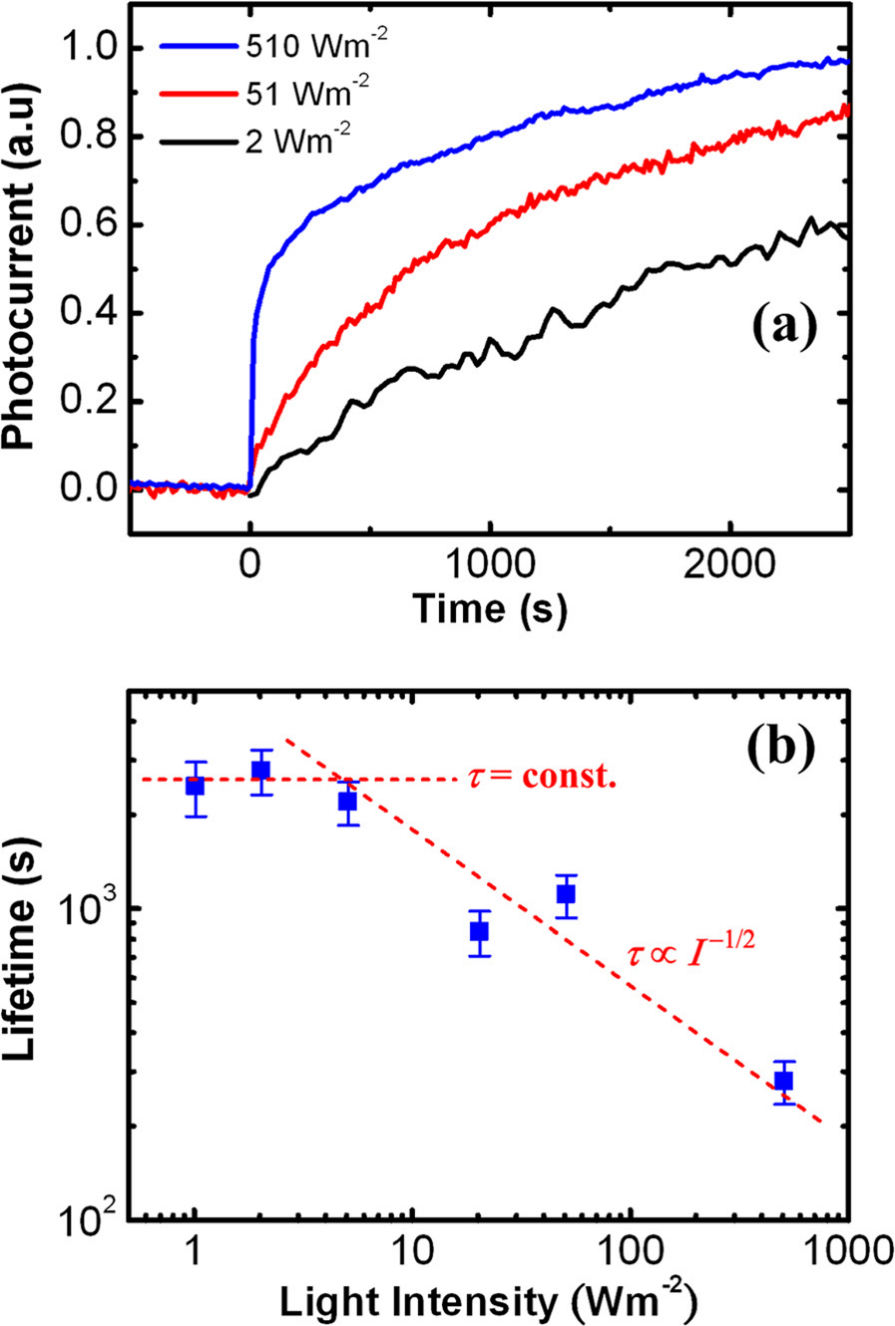
**Normalized photocurrent rise curves and fitted carrier lifetime versus intensity. (a)** The normalized photocurrent rise curves under inter-band excitation (*λ* = 325 nm) with selected intensity and **(b)** fitted carrier lifetime versus intensity measured at a bias of 0.1 V for the V_2_O_5_ NW with *d* = 800 nm and *l* = 2.5 μm.

The change of recombination behavior can be further verified by the power-dependent *τ* measurement. Figure 3a illustrates the normalized photocurrent rise curves under selected light intensity. The result shows that the rise time or photoresponse time increases with the decrease of power density. By fitting the photoresponse curves using stretched exponential function *i*_p_(*t*) = *i*_p0_ exp[-(*t*/*τ*)^*β*^], where *i*_p0_ is the steady-state photocurrent and *β* is the stretching factor smaller than unity; the dependence of *τ* on power density can be obtained and is depicted in Figure 3b. The result shows that the *τ* also follows the similar two-stage power dependence as *R* (or Γ), which further confirms the lifetime-dominant hole trapping PC mechanism in the V_2_O_5_ NWs.

According to literature reports, the photoconductivity of metal oxide semiconductor NWs, such as ZnO, SnO_2_, TiO_2_, and WO_3_, mostly follow a common oxygen-sensitized (OS) PC mechanism [36,37,43-45]. The mechanism is controlled by the interaction of foreign oxygen molecule and semiconductor in the near surface area. According to the OS model, the PC process includes four steps: (1) In the dark and in the atmospheric ambience, as oxygen plays a role of electron trap state in the metal oxide semiconductor surface, through oxygen adsorption, the electron is captured on the surface and creates negatively charged surface states (or oxygen ions) [O_2_(*g*) + *e*^-^ → O_2_^-^(*ad*)]. The effect induces an enhanced upward bending of the energy band at the surface. (2) Under light illumination, electron–hole pairs are generated [*hυ* → *e*^-^ + *h*^+^] and (3) subsequently separated by the surface electric field or band bending. (4) The excess holes are attracted by the surface and recombine with negative-charged oxygen on the surface [*h*^+^ + O_2_^-^(*ad*) → O_2_(*g*)]. The result leaves unpaired electrons with prolonged lifetimes, which is similar to the hole trapping effect in the bulk. Recombination can only take place when oxygen molecules re-adsorb on the surface as that in step 1.

**Figure 4:**
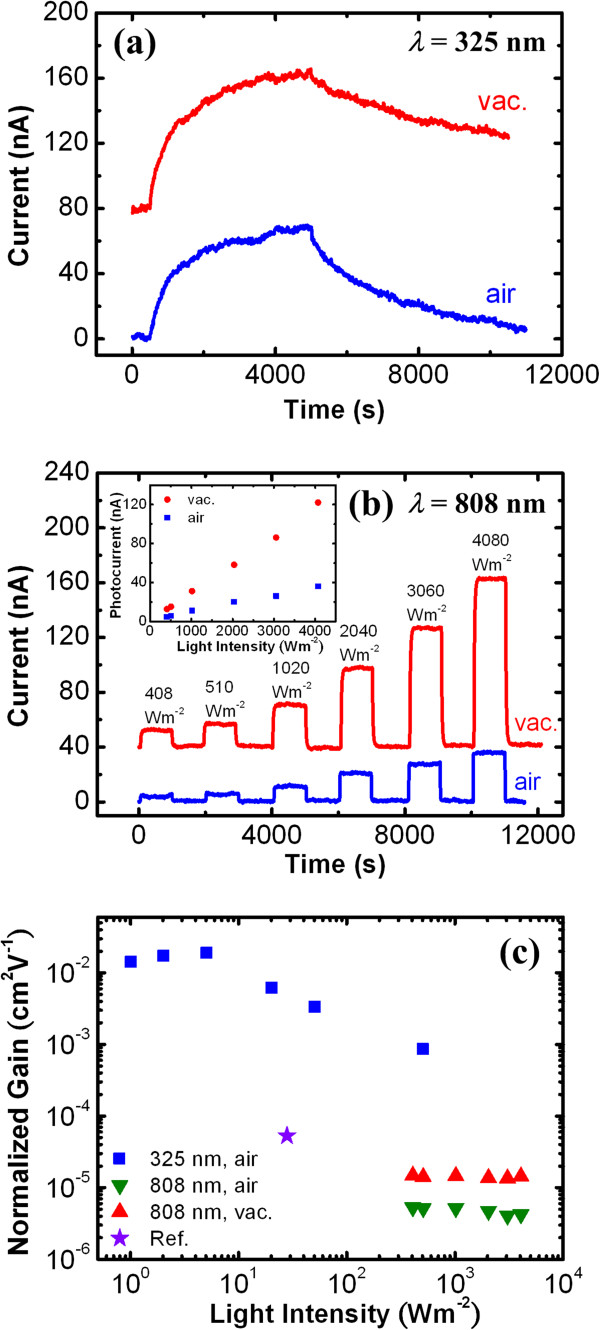
**Photoresponse curves under inter-and sub-bandgap excitations and calculated normalized gain versus intensity. (a)** The photoresponse curves under inter-bandgap excitation (*λ* = 325 nm) at *I* = 20 W m^-2^ in air and vacuum ambiences, **(b)** the photoresponse curves under sub-bandgap excitation (*λ* = 808 nm) at increasing *I* from 408 to 4,080 W m^-2^ in air and vacuum ambiences, and **(c)** the calculated normalized gain versus intensity at *λ* = 325 and 808 nm in air and vacuum ambiences for the V_2_O_5_ NW with *d* = 800 nm and *l* = 2.5 μm. The insert in **(b)** shows the photocurrent versus intensity plots at *λ* = 808 nm in air and vacuum.

By the aforementioned mechanism, the recombination rate and lifetime of the excess electron are governed by the oxygen adsorption rate. Therefore, the recombination rate of electrons can be highly reduced, and the *i*_p_ and *τ* can be enhanced while varying the ambience from air (oxygen-rich) to vacuum (oxygen-deficient). The ambience-dependent behavior of PC is the most direct measure to verify the surface-controlled PC mechanism in the metal oxide semiconductors. Accordingly, the environment-dependent photoresponse measurement for the V_2_O_5_ NWs was also performed. Figure 4a shows that the photoresponse curves measured in air and vacuum ambiences at *I* = 20 W m^-2^ of the V_2_O_5_ NW did not reveal any significant difference, which is distinct from the description of the OS mechanism. The V_2_O_5_ NW without surface effect under inter-band excitation actually is consistent with the bulk-dominant hole trapping mechanism observed by the power dependence study.

Although the photoconductivity of the V_2_O_5_ NWs has been confirmed to be dominated by the bulk under band-to-band (*λ* = 325 nm) excitation, the sub-bandgap excitation using the 808-nm wavelength (*E* = 1.53 eV) was also carried out to further characterize the layered 1D nanostructure. Figure 4b depicts the photoresponses under the sub-bandgap light illumination at different *I* and at *V* = 0.1 V in air and vacuum ambiences for the V_2_O_5_ NW with *d* = 800 nm and *l* = 2.5 μm. As the values of photoresponse at sub-bandgap excitation are much less than the inter-bandgap excitation, the *I* of the 808-nm wavelength was operated at a relatively high range of 408 to 4,080 W m^-2^. Under high-power condition, the sub-bandgap excitation generates an observable photoresponse and the *i*_p_ is linearly dependent on *I*. The *i*_p_ versus *I* curves in air and vacuum ambiences are also plotted in the inset of Figure 4b. The monotonic linear dependence of *i*_p_ and *I* is different from the two-stage power dependence for the band-to-band excitation in Figure 2b, implying the different PC mechanisms. The response time at a few seconds by 808-nm excitation is also much faster than that (*τ* > 100 s) by 325-nm excitation.

**Figure 5:**
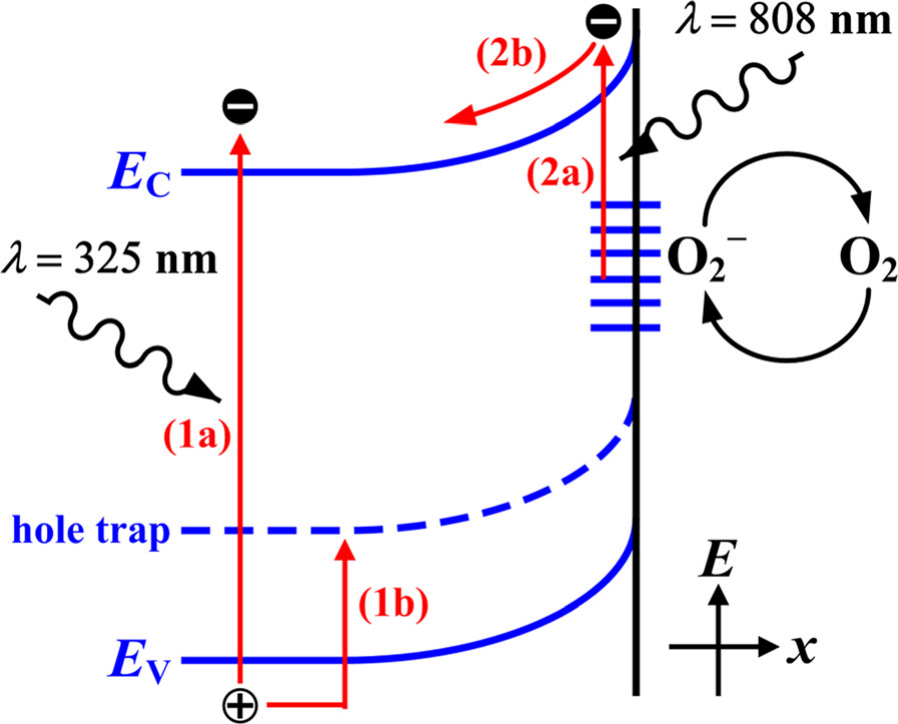
**The schematic PC processes for V**_**2**_**O**_**5 **_**NW.** Hole trapping effect in the bulk region by inter-bandgap excitation and oxygen sensitization effect in the surface by sub-bandgap excitation are illustrated respectively. Step (1a) electron–hole pair is generation by band-to-band excitation (*λ* = 325 nm) in the bulk; step (1b) hole is captured by the trap state leaving the unpaired electron with long lifetime. Step (2a) free electron is solely generated from the negatively charged surface state (or oxygen ion) by sub-bandgap excitation (*λ* = 808 nm); step (2b) electron attracted to the core with less recombination probability also exhibits prolonged lifetime. The recombination will only take place while foreign oxygen molecule recaptures electron on surface.

The more important difference is that the photoresponse under sub-bandgap excitation exhibits clear environment dependence. A similar behavior has also been observed by Tamang et al. [19]. The *i*_p_ in the vacuum is roughly three times higher than that in air. This observation is consistent with the OS mechanism in metal oxide semiconductors. Although the mechanism is usually described by the spatial separation of the electron–hole pair under above-bandgap excitation, the sub-bandgap light that excites electrons from the surface trap state to conduction band could result in a similar effect [46,47]. The schematic PC processes of hole trapping in the bulk and surface state excitations is shown in Figure 5. Although electron transition from the valence band to surface states may also generate a free hole which is able to recombine with oxygen ions and release trapped electrons leading to similar OS effect, the surface states are mostly occupied and negatively charged (i.e., the surface-adsorbed oxygen molecules are mostly ionized). The result indicates that the transition probability is rather low, which allows us to neglect the minor contribution. As light absorption only takes place at the surface, this could explain the very high power that is required to produce an observable photoresponse using the 808-nm excitation source.

To compare the PC efficiencies between the above- and below-bandgap excitations and between the V_2_O_5_ NWs grown by PVD and hydrothermal approaches, a new photoconductor parameter named normalized gain (Γ_n_) is adopted and discussed [45,48]. As the frequently used Γ is physically defined as the ratio of *τ* to transit time (*τ*_*t*_) between two electrodes of a device, i.e.,

$$\Gamma =\frac{\tau }{\tau_{t}},\;\text{and}\;\tau_{t}=\frac{l}{v},$$

where *v* is the carrier drift velocity which is equal to the product of mobility (*μ*) and applied electric field (*F*), i.e., *v* = *μF*, where $F=\frac{V}{l}$, Γ can be rewritten as $\Gamma =\frac{V}{l^{2}}\tau \mu$[29]. Accordingly, Γ depends on *l* and *V*. In terms of engineering application, photodetectors can be operated with high Γ by shortening *l* and increasing *V*. However, to objectively compare the intrinsic PC efficiency of the materials, the artificial factors have to be excluded.

Accordingly, we adopted the Γ_n_, physically defined as the product of *η*, *τ*, and *μ* (i.e., Γ_n_ = *ητμ*) [45,48]. As the *τμ* product is an intrinsic quantity determining photocarrier transport efficiency [42], for a constant *η*, Γ_n_ offers the same physical meaning as *τμ*, and its intrinsic property can exclude the effects of device dimension and experimental condition. In addition, Γ_n_ with the factor of *η* could take the real light absorption efficiency into account, whose importance has been demonstrated to further understand the PC process in 1D nanostructures [49]. Γ_n_ can be obtained by the following equation [45,48]:

(1) $$\Gamma_{n}=\Gamma \eta \frac{l^{2}}{V}\;=\frac{E}{q}\frac{i_{p}}{P}\frac{l^{2}}{V}.$$

The calculated Γ_n_ versus *I* using the data of Γ (Figure 2c) or *i*_p_ (Figure 2b) for the V_2_O_5_ NW measured at *V* = 0.1 V under 325-nm (*E* = 3.82 eV) and 808-nm (*E* = 1.53 eV) illuminations are illustrated in Figure 4c. One data point of hydrothermal-synthesized V_2_O_5_ NWs calculated according to the data in [2] (*E* = 2.76 eV) is also plotted for comparison.

After excluding the artificial contributions of *l* and *V*, the Γ_n_ of our PVD-grown V_2_O_5_ NWs at approximately 6 × 10^-3^ cm^2^ V^-1^ is two orders of magnitude higher than that (Γ_n_ ~ 5 × 10^-5^ cm^2^V^-1^) of the hydrothermal-synthesized ones for the similar *I* = 25 ± 5 W m^-2^. This result indicates the PVD-grown NWs exhibit a higher efficiency for photocarrier transport and photocurrent generation than the hydrothermal ones. The PVD (or thermal evaporation) approach usually provides better control for crystal growth, and the growth temperature at 550°C is also relatively high in comparison with that in the hydrothermal method (synthesis at 205°C). Accordingly, it is inferred that the higher PC efficiency (or Γ_n_) originated from a higher crystalline quality in this PVD-grown V_2_O_5_ nanostructure.

In addition, Figure 4c also shows that the Γ_n_ at 325-nm excitation is also much higher than that at 808-nm excitation. The optimal (saturation) Γ_n_ at *λ* = 325 nm is 1.7 ± 0.2 × 10^-2^ cm^2^ V^-1^ which is over three orders of magnitude higher than that (Γ_n_ = 4.7 ± 0.6 × 10^-6^ cm^2^ V^-1^) at *λ* = 808 nm in air ambience. The Γ_n_ enhanced in the vacuum can also be observed therein. The analysis quantitatively demonstrates the difference of PC efficiency induced by above- and below-bandgap excitations. As Γ_n_ linearly depends on *η* and *τ* and the volume for optical absorption (or *η*) of the bulk by inter-bandgap excitation is much higher than that of the surface under sub-bandgap excitation, it is proposed that *η* plays an important role on the Γ_n_ difference for the wavelength-dependent PC. The relatively long photoresponse time (or *τ*) could also contribute to the higher Γ_n_ under inter-bandgap (325 nm) excitation.

Finally, it is noted that the PC mechanism based on the small polaron hopping transport has been proposed by Lu et al. [21]. The very short lifetimes in the range of 1 to 1,000 μs are usually one of the criteria to manifest the polaron hopping mechanism. However, the typical lifetimes in this study either under 325 or under 808 excitation are at the orders of magnitude from seconds to hundred seconds, which is at least three orders of magnitude higher than the relaxation time of small polaron. The substantial difference could allow us to explain the PC mechanism on the basis of the conventional band conduction model (as shown in Figure 5) for monocrystalline semiconductors. The free electron-dominant conduction mechanism could also offer a probable explanation for the relatively higher *σ* in the PVD-grown V_2_O_5_ NWs in comparison with the literature data of which hopping is the dominant factor for charge conduction [23,24].

## Conclusions

Photoconductivities of the PVD-grown V_2_O_5_ NWs with monocrystalline orthorhombic structure have been investigated. In addition to the device performance, the PVD-grown V_2_O_5_ NWs exhibit two orders of magnitude higher PC efficiency (or Γ_n_) than their hydrothermal-synthesized counterparts. In addition, the PC mechanism has also been studied by the power, environment, and wavelength-dependent measurements. Both the bulk-controlled (hole trapping effect) and surface-controlled (oxygen-sensitization effect) PC mechanisms have been observed under above- and below-bandgap excitations, respectively. Understanding of the transport properties in this layered V_2_O_5_ 1D nanostructure could enable us to design the electronic, optoelectronic, and electrochemical devices by a more efficient way.

## Competing interests

The authors declare that they have no competing interests.

## Authors’ contributions

RSC designed the experiments, analyzed the data, proposed the model, and drafted the manuscript. WCW and CHC carried out experimental measurements. HPH participated in the result discussion. LCT and YJC carried out material growth. All authors read and approved the final manuscript.
